# DNA damage response genes as biomarkers of therapeutic outcomes in acute myeloid leukemia patients

**DOI:** 10.1038/s41375-024-02269-9

**Published:** 2024-05-11

**Authors:** Adam Karami, Tomasz Skorski

**Affiliations:** 1 Fels Cancer Institute for Personalized Medicine, Lewis Katz School of Medicine, Temple University, Philadelphia, PA 19140 USA; 2 Department of Cancer and Cellular Biology, Lewis Katz School of Medicine, Temple University, Philadelphia, PA 19140 USA; 3 Nuclear Dynamics and Cancer Program, Fox Chase Cancer Center, Philadelphia, PA USA

**Keywords:** Cancer genomics, Cancer therapy

## To the Editor:

Current AML therapeutics induce DNA damage and/or modulate DNA damage response (DDR) directly or indirectly [1, 2]. Recent reports analyzed large datasets to predict the response of AML patients to the treatment [3–5]. The combinatorial mutational events (e.g., *NRAS, TP53, TET2, IDH1* and/or *NPM1*) as well as single gene expression levels (e.g., *PEAR1*) were implicated as potential biomarkers of the clinical outcome. These genes however, except *TP53*, are not involved in DDR.

**Fig. 1:**
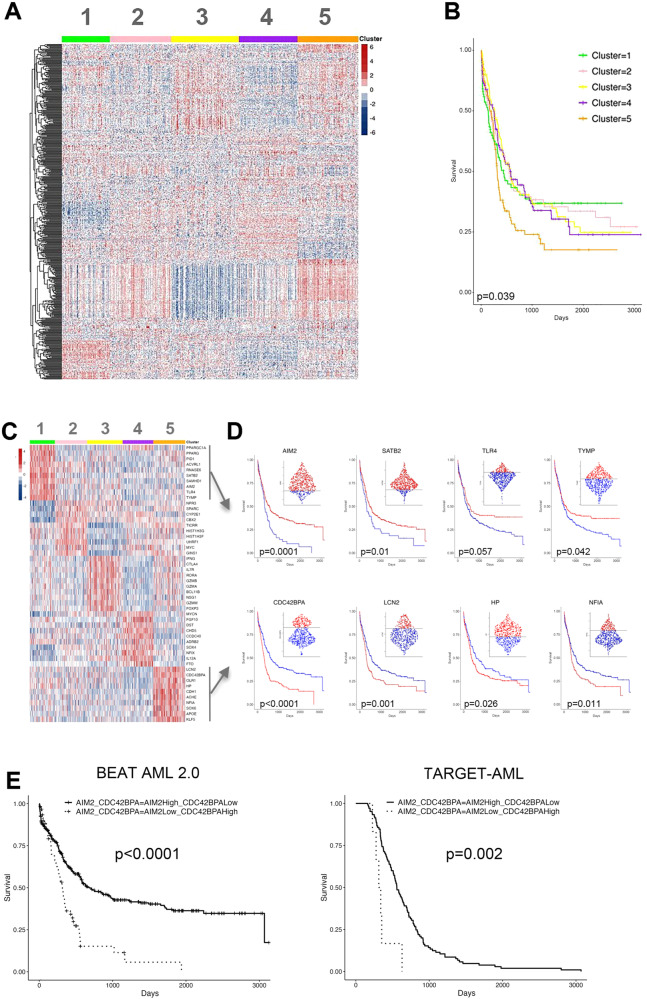
DDR genes as biomarkers predicting survival of AML patients. **A** Consensus clustering of AML subjects in the BEAT AML 2.0 dataset into 5 groups with varying DDR transcriptomic signatures. **B** Kaplan-Meier plot showing the survival of the 5 groups. **C** Top 10 DDR biomarker genes with the highest expression in each cluster. **D** Kaplan-Meier plots of the difference in survival between subjects with high versus low scaled and normalized values of the DDR biomarker genes for clusters 1 and 5. High versus low cut points were determined via Thiele and Hirschfield’s method to find an optimal outcome-based cut point, survival. **E** The combined values of AIM2 and CDC42BPA are a powerful biomarker for survival in BEAT AML 2.0 and TARGET-recurrent AML databases.

Here, we employed a list of 1800 DDR-related genes [6] to interrogate a cohort of 612 AML patients (612 specimens) from waves 1 to 4 of the training BEAT AML 2.0 dataset [3] to determine the correlations between DDR gene expression and therapeutic outcomes. Only 500 genes were chosen for actual analysis based on their median absolute deviation across the dataset. This was done to reduce background noise in the dataset and to only use genes with high variation across subjects. Consensus clustering separated AML subjects into 5 groups with varying DDR transcriptomic signatures (Fig. 1A). Kaplan-Meier analysis of the survival profiles of the 5 groups revealed cluster 1 displaying the best prognosis while cluster 5 with the worst (Fig. 1B). This prediction was validated by detecting higher frequency of parameters associated with poor prognosis (treatment-refractory cases, prior-MDS, prior-MPN and *TP53* mutation) in cluster 5 when compared to cluster 1 (Supplementary Fig. S1). Except consensus sex (more males in cluster 5 vs cluster 1) no other distribution demographics (race, ethnicity, and age) were different in cluster 5 compared to cluster 1 (Supplementary Fig. S2).

The top 10 DDR biomarker genes with the highest expression in each cluster are indicated in Fig. 1C. Individual gene analysis revealed 4 genes in cluster 1 and cluster 5 which enhanced expression correlated with good (*AIM2, SATB2, TYMP* and *TLR4* in cluster 1) and poor (*CDC42BPA, LCN2, NF1A* and *HP* in cluster 5) prognosis (Fig. 1D). The most significant cluster marker genes discriminating between good and poor survival were *AIM2* (cluster 1 biomarker, high values denoted good prognosis) and *CDC42BPA* (cluster 5 biomarker, high values denoted bad prognosis).

Absent in melanoma 2 (AIM2) detects DNA in the cytosol and assembles an inflammasome, which activates caspase-1 and pro-inflammatory cytokines leading to pyroptosis, an inflammatory form of cell death [7]. Thus, AIM2 can facilitate pyroptosis in AML cells which accumulate cytosolic DNA during treatment, leading to accelerated elimination of AML cells and favorable prognosis.

CDC42 binding protein kinase alpha (CDC42BPA*)* also known as th*e* myotonic dystrophy protein kinase-like alpha (MRCKα) is required for TP53-dependent autophagy [8]. Autophagy protects leukemia cells during chemotherapy by providing energy and facilitating proliferation through the supply of essential components such as amino acids and nucleotides. In addition, CDC42BPA has been also implicated in regulation the sensitivity of high-grade serous ovarian carcinoma and glioblastoma cells to chemotherapy and radiotherapy and/or in tumor cell growth [9]. Thus, CDC42BPA might regulate chemotherapy resistance of AML cells and inhibitors of the kinase may have therapeutic application in a cohort of high *CDC42BPA* expressors [9]. Of note, while *TP53* expression did not alter between high and low *CDC42BPA* expressors, *TP53* mutations were found in 19.9% high *CDC42BPA* expressors, compared to only 3.7% of the low *CDC42BPA* expressors. Therefore, higher frequency of *TP53* mutants among high *CDC42BPA* expressors might also contribute to worse survival.

Remarkably, combined values of the expression of *AIM2* and *CDC42BPA* are extremely powerful predictor for survival in the training BEAT AML 2.0 database (Fig. 1E). AML patients with high values of *AIM2* and low values of *CDC42BPA* expression displayed the best prognosis, whereas these with low values of *AIM2* and high values of *CDC42BPA* had the worst prognosis. The discovery of biomarker values of *AIM2* and *CDC42BPA* as predictors of survival were validated using TARGET-recurrent AML dataset from 427 patients (Fig. 1E).

AML cells accumulate high numbers of DNA double-strand breaks (DSBs), the most lethal of all DNA lesions resulting from altered metabolism [10, 11] and induced by therapeutic approaches [1]. DSBs are highly deleterious, with a single unrepaired DSB being sufficient to trigger cell death [12]. To repair numerous DSBs AML cells may activate the DSB repair mechanisms involving RAD51-mediated homologous recombination (HR), DNA-PK -mediated non-homologous end-joining (NHEJ) and DNA polymerase theta (Polθ, encoded by *POLQ* gene)-dependent microhomology-mediated end-joining (TMEJ) [13] Therefore, we also tested if expression levels of DSB repair genes had a survival prognostication value.

**Fig. 2:**
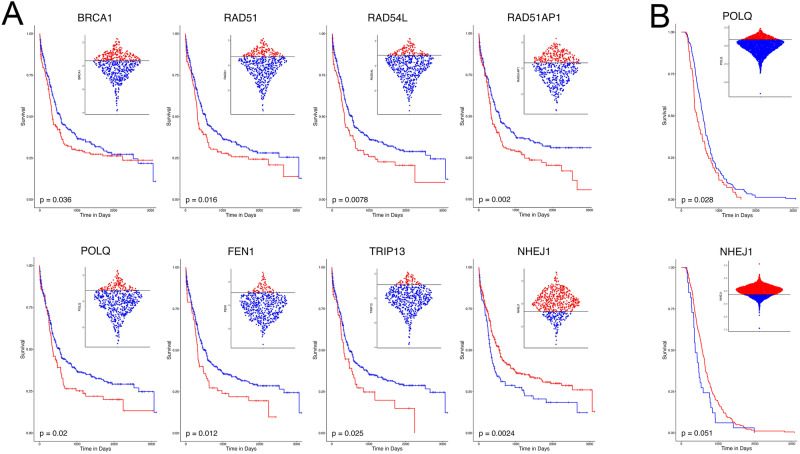
Expression of DSB repair genes predicts survival of AML. **A**, **B** Kaplan-Meier plots showing the difference in survival between subjects with high versus low scaled and normalized values of the DSB repair genes in the BEAT AML 2.0 (**A**) and TARGET-recurrent AML (**B**) datasets. High versus low cut points were determined via Thiele and Hirschfield’s method to find an optimal outcome-based cut point, survival.

Analysis of training BEAT AML 2.0 database revealed that upregulation of several genes involved in RAD51-mediated HR (*BRCA1, RAD51, RAD54L* and *RAD51AP1*) and Polθ-dependent TMEJ (*POLQ, FEN1* and *TRIP13*) were associated with worse prognosis (Fig. 2A). Conversely, patients with elevated expression of *NHEJ1* gene (non-homologous end-joining factor 1), a member of NHEJ displayed better prognosis. Remarkably, the biomarker prognosticator values of *POLQ* and *NHEJ1* gene expression were validated in TARGET-recurrent AML dataset (Fig. 2B).

In conclusion, using DDR genes as probes we identified novel biomarkers for AML prognostication: co-expression of *AIM2* and *CDC42BPA* genes and sole expression of *POLQ*. Overexpression of Polθ confers cellular resistance to various genotoxic cancer therapies (ionizing radiation, genotoxic chemotherapy drugs) [14]. The discovery that *POLQ* is a key prognostic biomarker in AML may have a therapeutic value because cancer cells, including AML cells overexpressing Polθ protein are hypersensitive to Polθ inhibitors which are currently in clinical trials against solid tumors [10, 15].

### Supplementary information

Supplemental Methods and Results
