# Supplementary material for: DNA damage response genes as biomarkers of therapeutic outcomes in acute myeloid leukemia patients
Source: Leukemia. 2024 May 11;38(6):1407–10. doi: 10.1038/s41375-024-02269-9 (PMC11147752; doi:10.1038/s41375-024-02269-9)
Supplement: Supplementary file 1 — Supplemental Methods and Results [file 41375_2024_2269_MOESM1_ESM.pdf]

Supplemental Materials and Methods

Training dataset
For the training dataset, we used clinical and expression data from the BEAT AML 2.0 database (1) (https://datacatalog.ccdi.cancer.gov/dataset/Vizome-BEATAML2.0). The expression data has been quantile normalized to account for multiple subject waves, and the data was further gene wise z-score scaled.

Gene selection
From a list of 1800 DDR-related genes (2), 500 were chosen based on their median absolute deviation across the dataset. This was done to reduce background noise in the dataset and to only use genes with high variation across subjects.

Consensus clustering
Consensus clustering on the scaled dataset with the selected genes was done with the R library ConsensusClusterPlus (https://academic.oup.com/bioinformatics/article/26/12/1572/281699). Briefly, clustering was done iteratively taking into account the Pearson correlation distances. 20 k values were calculated with 1000 resamples. Resampling was done with 80% of the subjects and 100% of the 500 chosen genes. The clustering algorithm employed for this purpose was PAM (partitioning around medoids or k-medoids). The final choice for the number of k clusters was determined by the k with the highest silhouette score (5 clusters at 8.6). Figures for the chosen k and its clustering statistics can be found in Supplemental Figure S3.

Gene cut point determination
Gene cut points to separate subjects into high and low expression groups used the R survminer (https://CRAN.R-project.org/package=survminer), which optimizes cut points based on an outcome variable. For our purposes, we chose subject survival as the clinical outcome and the normalized scaled gene wise expression values to fit the algorithm. To determine the robustness of each gene as a prognosticator, the cut points calculated in the training dataset (BEAT-AML) were also used in the validation TARGET-AML dataset. The code for cutpoint determination, sample segregation, and survival curve generation can be found on https://github.com/alkarami/LAML_Review_2024/tree/main.

Survival analysis
Kaplan-Meier curves were calculated using the R library survival (https://cran.r-project.org/web/packages/survival/vignettes/survival.pdf). For BEAT-AML, the curves were fitted with days to last follow-up and the vital status at follow-up - censoring the subjects still alive at the last check-in. Cox proportional hazard ratios were also done for each gene using the same variables. Kaplan-Meier curves were plotted with the R library survminer (https://cran.r-project.org/web/packages/survminer/index.html), with p-values adjusted with Benjamini-Hochberg correction for multiple testing.

Validation dataset
Expression and clinical data from TARGET-AML (https://www.cancer.gov/ccg/research/genome-sequencing/target/studied-cancers/acute-myeloid-leukemia) were obtained from the GDC portal via the TCGA biolinks R library (3). The dataset has already been normalized, and gene wise z-score scaling was applied to the 500 genes chosen and used in the BEAT AML 2.0 dataset. For each gene, cut points calculated in the BEAT AML 2.0 dataset were used to classify high and low expression groups. The curves were fitted with days to death instead as the dataset does not have days to last follow-up available.

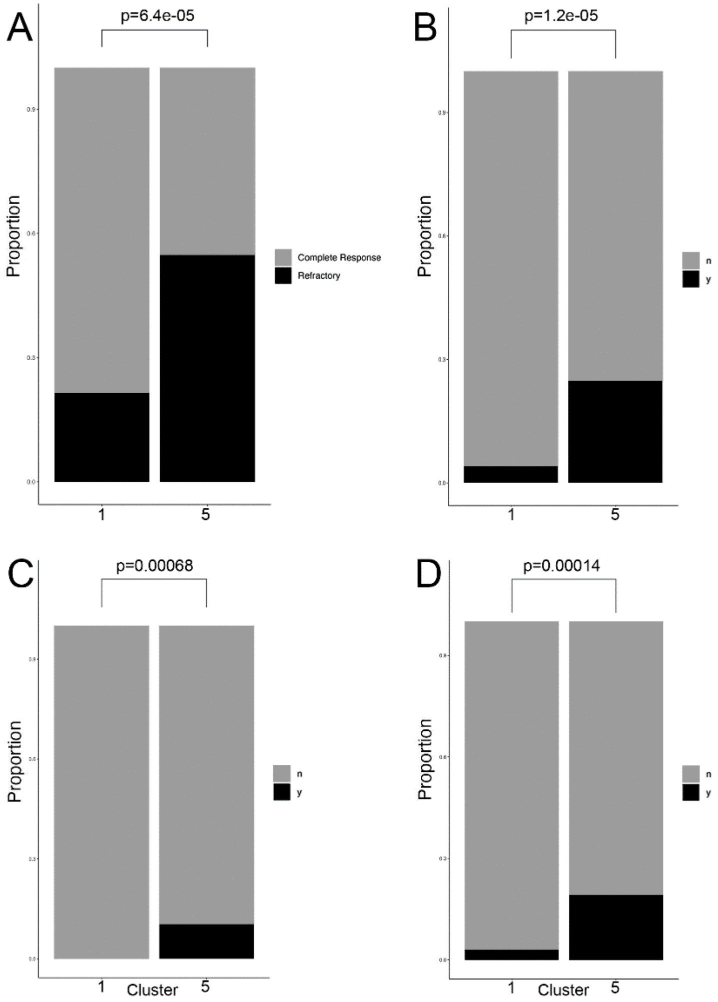

Supplemental Figure S1. Comparisons of clinical variables (**A**. response to induction therapy, **B**. prior MDS, **C**. prior MPN, **D**. *TP53* mutation) between clusters 1 and 5, shown as the proportions of values in the two clusters (4-7). Fisher's exact tests were done to determine the significance of the difference found between the clusters, with the p-values shown for each comparison.

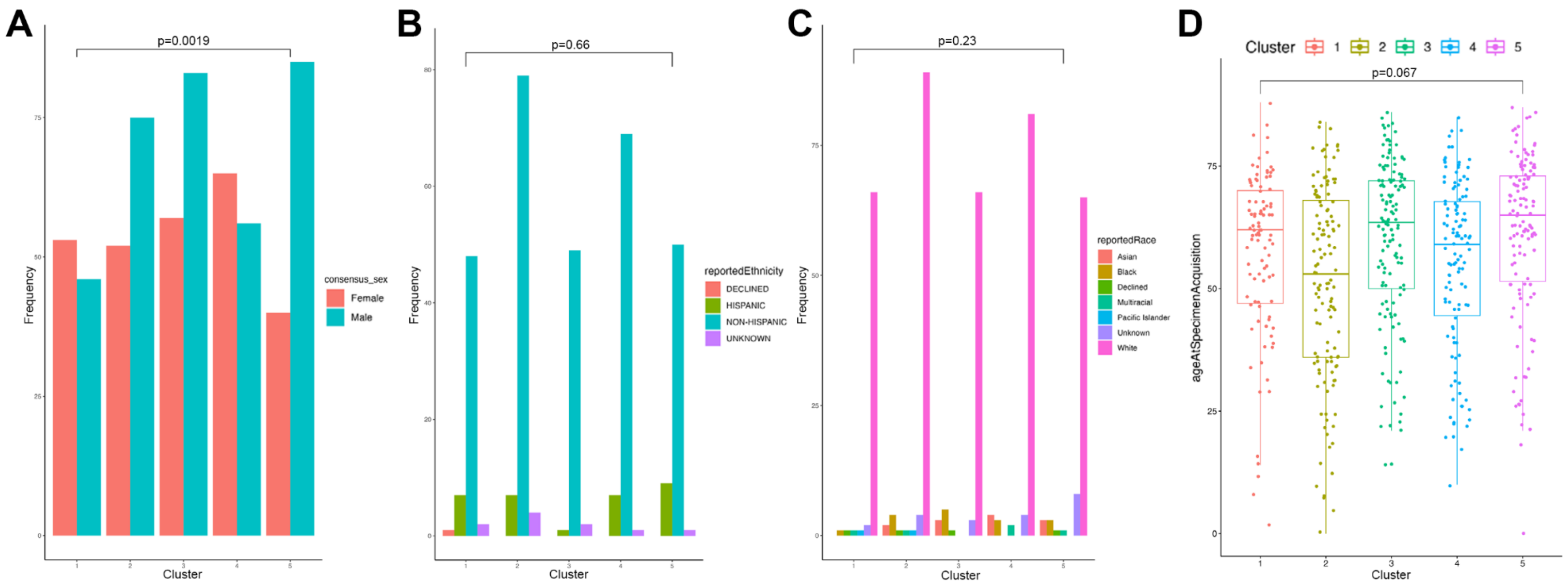

Supplemental Figure S2: Demographic clinical variables for the 612 BEAT-AML subjects in the training dataset along with statistical comparisons of subgroups 1 and 5**. A.** Consensus sex distribution for each cluster. **B.** Reported ethnicity distribution for each cluster. **C.** Reported race distribution for each cluster. **D.** Age at specimen acquisition distribution for each cluster. P value was calculated by log rank test.

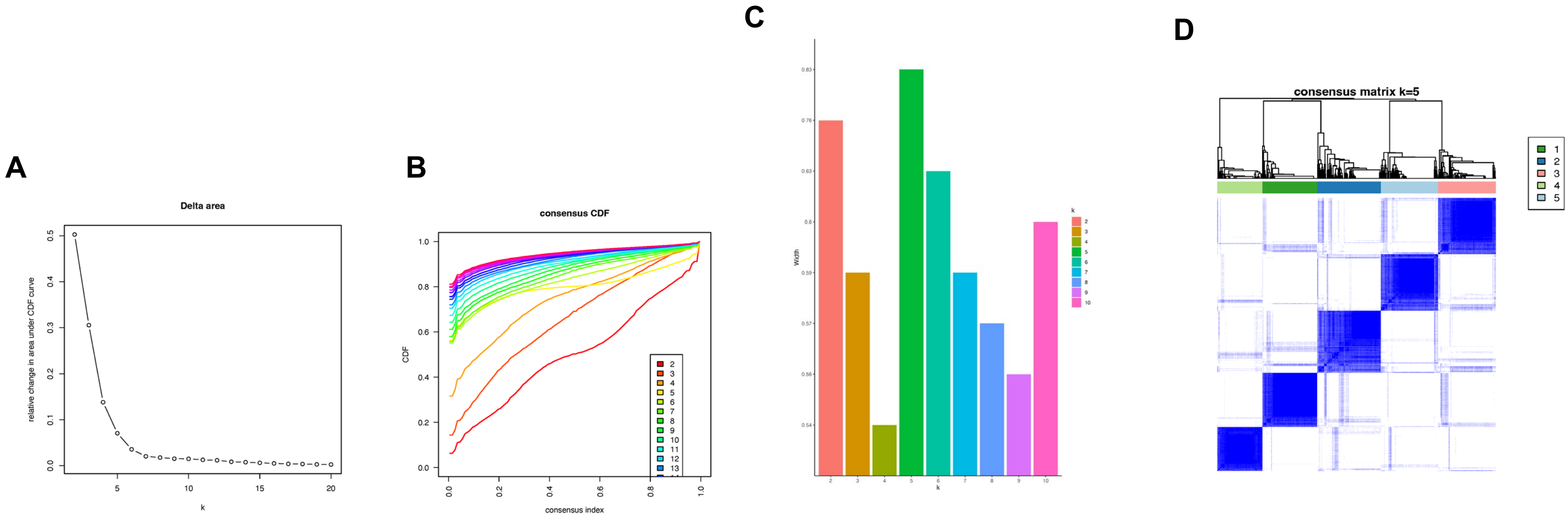

Supplemental Figure S3: Cluster optimization parameters for the choice of k number of clusters. **A.** Delta area plot for the tested k clusters. **B.** Consensus CDF plot for the tested k clusters. **C.** Average silhouette widths for the first k clusters tested, showing the highest average width for k = 5 at 8.3. **D.** Consensus matrix for the chosen k = 5.

References

1. Bottomly D, Long N, Schultz AR, Kurtz SE, Tognon CE, Johnson K*, et al.* Integrative analysis of drug response and clinical outcome in acute myeloid leukemia. Cancer cell **2022**;40:850-64.e9
2. Gogola E, Duarte AA, de Ruiter JR, Wiegant WW, Schmid JA, de Bruijn R*, et al.* Selective Loss of PARG Restores PARylation and Counteracts PARP Inhibitor-Mediated Synthetic Lethality. Cancer cell **2018**;33:1078-93.e12
3. Colaprico A, Silva TC, Olsen C, Garofano L, Cava C, Garolini D*, et al.* TCGAbiolinks: an R/Bioconductor package for integrative analysis of TCGA data. Nucleic acids research **2016**;44:e71
4. Jabbour E, Ghanem H, Huang X, Ravandi F, Garcia-Manero G, O'Brien S*, et al.* Acute myeloid leukemia after myelodysplastic syndrome and failure of therapy with hypomethylating agents: an emerging entity with a poor prognosis. Clin Lymphoma Myeloma Leuk **2014**;14:93-7
5. Mannelli F. Acute Myeloid Leukemia Evolving from Myeloproliferative Neoplasms: Many Sides of a Challenging Disease. J Clin Med **2021**;10
6. Tasaki T, Yamauchi T, Matsuda Y, Takai M, Ookura M, Lee S*, et al.* The response to induction therapy is crucial for the treatment outcomes of elderly patients with acute myeloid leukemia: single-institution experience. Anticancer Res **2014**;34:5631-6
7. Qin G, Han X. The Prognostic Value of TP53 Mutations in Adult Acute Myeloid Leukemia: A Meta-Analysis. Transfus Med Hemother **2023**;50:234-44
